# The Conservation Value of Traditional Rural Landscapes: The Case of Woodpeckers in Transylvania, Romania

**DOI:** 10.1371/journal.pone.0065236

**Published:** 2013-06-19

**Authors:** Ine Dorresteijn, Tibor Hartel, Jan Hanspach, Henrik von Wehrden, Joern Fischer

**Affiliations:** 1 Faculty of Sustainability, Leuphana University Lueneburg, Lüneburg, Germany; 2 Center for Methods, Leuphana University Lueneburg, Lüneburg, Germany; 3 Department of Environmental Sciences, Sapientia University, Cluj-Napoca, Romania; Umea University, Sweden

## Abstract

Land use change is a major threat to global biodiversity. Forest species face the dual threats of deforestation and intensification of forest management. In regions where forests are under threat, rural landscapes that retain structural components of mature forests potentially provide valuable additional habitat for some forest species. Here, we illustrate the habitat value of traditional wood pastures for a woodpecker assemblage of six species in southern Transylvania, Romania. Wood pastures are created by long-term stable silvo-pastoral management practices, and are composed of open grassland with scattered large, old trees. Because of their demanding habitat requirements, woodpeckers share habitat with many other bird species, and have been considered as possible indicator species for bird species diversity. We first compared woodpecker assemblages between forests and wood pastures. Second, we grouped features of wood pastures into three spatial contexts and addressed how these features related to the occurrence of three woodpecker species that are formally protected. Woodpecker species composition, but not the number of species, differed between forests and wood pastures, with the green woodpecker occurring more commonly in wood pastures, and the lesser spotted woodpecker more commonly in forests. Within wood pastures, the intermediate context (especially surrounding forest cover) best explained the presence of the grey-headed and middle spotted woodpecker. By contrast, variables describing local vegetation structure and characteristics of the surrounding landscape did not affect woodpecker occurrence in wood pastures. In contrast to many other parts of Europe, in which several species of woodpeckers have declined, the traditional rural landscape of Transylvania continues to provide habitat for several woodpecker species, both in forests and wood pastures. Given the apparent habitat value of wood pastures for woodpeckers we recommend wood pastures be explicitly considered in relevant policies of the European Union, namely the Habitats Directive and the EU Common Agricultural Policy.

## Introduction

Human-induced landscape change poses a major threat to global biodiversity [1], [2]. Forest species face the dual threat of deforestation and intensification of forest management. Woodpeckers are especially sensitive to these changes because they require large home ranges and depend on large trees for nesting and dead wood for foraging [3]. Consequently, changes in forest structure and cover have caused woodpecker declines worldwide [4]–[6]. Because of their demanding habitat requirements, woodpeckers share habitat requirements with many other bird species. Therefore, woodpeckers have been considered as potential indicator species for bird species diversity [7].

In Europe, six out of ten species of woodpeckers are protected under the EU Birds Directive Annex I. While several woodpecker species have declined in Western Europe, Eastern European woodpecker assemblages have remained diverse and stable due to the persistence of large forest tracts [8]. However, Eastern European forest landscapes are increasingly coming under pressure from more intensive and widespread logging operations [9]. Against this background, it is important to understand to what extent the landscape context surrounding forest patches could provide complementary habitat for woodpeckers in Eastern Europe. Many agricultural landscapes in Eastern Europe are characterized by low-intensity subsistence farming that still contain semi-natural vegetation cover, including transitional elements between forest patches such as scattered trees [10]. Such relatively complex landscapes have the potential to support high biodiversity. However, the links between biodiversity and land use are still relatively poorly understood in Eastern Europe [10]. Mikusinski and Angelstam [8] proposed that rural landscapes retaining remnant structures of natural forests could provide valuable additional habitat for species otherwise confined to forests, but little work has specifically tested this claim to date.

One of the most intact traditional rural landscapes in lowland Europe occurs in southern Transylvania (Romania). In this area, traditional wood pastures are of particular interest in terms of their potential habitat value for woodpeckers. Many wood pastures in southern Transylvania are several centuries old; they are composed of open grassland with scattered trees and result from ancient silvo-pastoral management practices. Wood pastures bring together three ecologically important components. First, scattered trees have a disproportionate ecological value, that is their effect on ecosystem functioning is disproportionately large relative to the small area occupied by an individual tree, both locally [11] and at a landscape scale [12], [13]. Second, many wood pastures contain large and old trees [14], [15], and therefore incorporate structural attributes that support biodiversity elements typical of old-growth forests [16]. Third, wood pastures can provide habitat for both open-country and forest species. Despite their high potential to support biodiversity, the ecological importance of wood pastures, especially in Eastern Europe, remains poorly understood (but see e.g. [17]–[19]). However, existing evidence from Western Europe suggests that wood pastures can provide habitat for a range of bird species including woodpeckers and secondary cavity-nesting birds [15], [20].

Here, we document the habitat value of wood pastures for an assemblage of six woodpecker species in Transylvania, Romania. Our specific objectives were to (1) compare woodpecker communities between wood pastures and forests; and (2) assess which features of a wood pasture are particularly important for different woodpecker species. Our findings highlight the conservation value of wood pastures in traditional rural landscapes in Eastern Europe.

## Methods

### Ethics Statement

All necessary permits were obtained for the described study. Permission to survey woodpeckers within the EU *Natura 2000* network was granted by Progresul Silvic, the organisation officially entrusted with the custody of the protected area by the Romanian government. The survey procedure, including the use of playback calls for protected species, was cleared by the ethics committee of Leuphana University Lueneburg.

**Figure 1:**
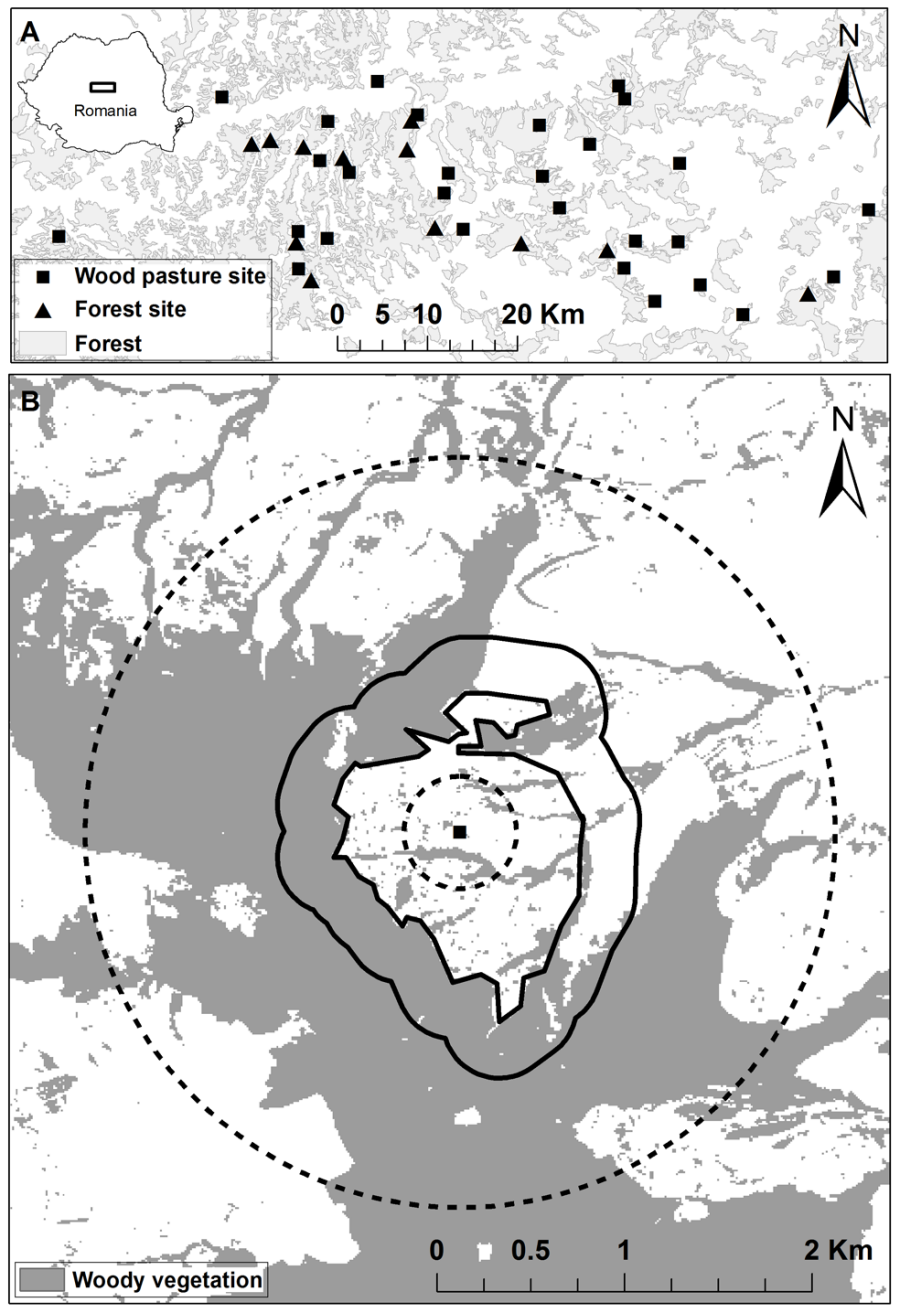
Study area and design. A) The location of the study area in southern Transylvania, Romania, and location of the 12 forest and 28 wood pasture sites. B) Example of a survey site, showing the three different landscape extent groups considered in the analyses. The small dashed circle (a 300 m radius around the survey point) represents the local context; solid lines represent the intermediate context (a 300 m buffer from the border of the wood pasture); and the large dashed circle represents the broader landscape context (a 2000 m radius around the survey point).

Woodpeckers were surveyed in 28 wood pastures and 12 forests in Southern Transylvania, Romania (Fig. 1a). We sampled a larger number of wood pastures than forests because forests are relatively homogenous, whereas wood pastures differ substantially in structural elements and adjacent forest cover. Furthermore, we were especially interested in which features of a wood pasture affected woodpecker presence and thus chose more sites to comprehensively cover existing gradients within wood pastures. Wood pastures were chosen on the basis of availability and access, and to cover a gradient in surrounding forest cover (min. = 3.3%; max. = 96.8%; mean ± SE = 59.7±4.8%). Within each wood pasture we choose one survey site, located approximately in the centre of the wood pasture, for woodpecker point counts. Forests were chosen on the basis of accessibility, and each forest site was located randomly at a distance of at least 600 m from the forest edge. Wood pastures and forests differed in tree species composition: forests were dominated by oak (*Quercus robur* and *Q. petrea*), hornbeam (*Carpinus betulus*) and beech (*Fagus sylvatica*), whereas wood pastures were dominated by oak and had more fruit trees (mainly pear, *Pyrus pyraster*) (Table 1). Trees were larger (and typically older) and occurred at a lower density in wood pastures than in forests (Table 1).

**Table 1:** Habitat characteristics (mean± SE) of the two surveyed habitat types: forests (n = 12) and wood pastures (n = 28).

	Forest	Wood pasture
Habitat variables; mean ± SE		
Number of trees	1345±255.89	16.54±1.77
Median DBH	25.97±2.69	76.52±4.86
Proportion of oak	0.20±0.03	0.63±0.04
Proportion of hornbeam	0.44±0.05	0.12±0.03
Proportion of beech	0.33±0.07	0.05±0.02
Proportion of fruit trees	0.003±0.001	0.15±0.03

The number of trees was calculated as the number of trees in 2 ha; median dbh (cm) was calculated as the mean of the medians measured within 2 ha and between 80 and 300 m; the proportion of a tree species was calculated as the mean of the proportion of a species in 2 ha and between 80 and 300 m.

We surveyed the six most common woodpecker species, namely great spotted woodpecker (*Dendrocopos major*), middle spotted woodpecker (*D. medius*), lesser spotted woodpecker (*D. minor*), green woodpecker (*Picus viridis*), grey-headed woodpecker (*P. canus*), and black woodpecker (*Dryocopus martius*). The middle-spotted woodpecker, grey-headed woodpecker and black woodpecker are protected under the EU Birds Directive Annex I. We did not include the Syrian woodpecker (*Dendrocopos syriacus*) and white-backed woodpecker (*Dendrocopos leucotos*) into our study because they are very rare in our study area, and we did not encounter these species during our surveys. We used unlimited point counts enhanced by the use of playbacks of woodpecker drummings and/or calls with an audible range of approximately 150 m. Following 5 minutes of initial listening, playbacks were used in a sequence from smallest to largest species [21]. For each species, a sequence of drummings and/or calls was played three times for 15 seconds, with 15 seconds of silence between playbacks and 1.5 minutes of silence between species. All sites were surveyed three times during appropriate weather conditions between 10 March and 6 April 2012.

To characterize the structure of sites, we recorded the species and diameter at breast height (dbh) of: (1) within 80 m of the survey point ( = 2 ha), all trees in wood pastures (1 to 34 trees) or 50 trees in forests (selected randomly in a spiral from the survey point to the edge of 2 ha); and (2) in four strip transects of 10 m width between 80 and 300 m from the survey point in the four cardinal directions (N, S, E, W). In case that no or too few trees were found on a transect we measured between one and four trees close to the transect. For forest sites, we estimated tree density in 2 ha by doubling the count of all trees in two opposite quarters of a 2 ha circle; in wood pastures we counted all trees within 2 ha around the survey point.

All analyses were performed on presence/absence data, pooled across the three repeats, and implemented in the ‘R’ environment [22]. First, we assessed differences in woodpecker species composition and the number of species between forest sites and wood pastures. We conducted nonmetric multi-dimensional scaling based on Sørensen dissimilarity to assess the differences in woodpecker composition between forests and wood pastures using the R package ‘Vegan’. We used analysis of similarity (ANOSIM; Sørensen dissimilarities) with 1000 permutations to test for differences in woodpecker composition between forests and wood pastures. In a last step, we modelled the number of woodpecker species as a function of site type using a generalized linear model with Poisson error structure. Although using playbacks and three repeat surveys presumably decreased the incidence of false absences, they cannot be ruled out. This is especially the case for the species that were relatively uncommon (number of times a species was observed at least twice compared to total number of sites present: great spotted woodpecker: 21/33; middle spotted woodpecker: 7/15; lesser spotted woodpecker: 1/9; grey-headed woodpecker: 9/22; green woodpecker: 23/30; black woodpecker: 4/10). Importantly, however, we are confident that detectability did not differ between forests and wood pastures. Potential differences in visibility between forests and wood pastures were of minor importance because: (1) most woodpeckers were identified using calls; and (2) trees did not yet have leaves and visibility in forests was therefore good.

Second, we assessed which features of a wood pasture were important for different species of woodpeckers. The response of the three species protected under the EU Birds Directive to environmental variables was analysed using generalized linear models with a binomial error structure. We did not include the forest sites in these analyses because their habitat characteristics were very different from wood pastures (Table 1), and we were specifically interested in which features of wood pastures affected woodpecker occurrence. We did not analyse the three unprotected species because they occurred too rarely or too frequently to be modelled (site occurrence of great spotted woodpecker: 23/28; green woodpecker: 26/28; lesser spotted woodpecker: 5/28).

We hypothesized that different site-specific and landscape variables might influence woodpecker presence, and we therefore grouped our explanatory variables as follows: local context (L) represented by a circle with a 300 m radius around a site; intermediate context (I) represented as an irregular buffer of 300 m around a particular wood pasture; and broader landscape context (B) represented by a circle with a 2000 m radius around a site (Fig. 1b). Scales were selected both to match the scale of the wood pastures and to be ecologically meaningful to woodpeckers.

Local variables included the proportion of oak, median dbh, and percent woody vegetation cover. Woodpeckers generally prefer older trees for nesting, with several species showing a particular preference for oak [23], [24]. Proportion of oak was estimated by calculating the mean of the proportion of oak in 2 ha and between 80 and 300 m, and median dbh was estimated by calculating the mean of the median diameters measured within 2 ha and between 80 and 300 m. Percent woody vegetation was derived from a supervised classifications of the panchromatic channels of SPOT 5 data (©CNES 2007, Distribution Spot Image SA) using a support vector machine algorithm [25].

Intermediate context variables were related to specific structures of the wood pasture and included the area of the wood pasture and percent woody vegetation cover within a 300 m buffer from the edge of the wood pasture (as a measure of how much of the perimeter of a given wood pasture was adjacent to forest). Adjacency to forest cover was considered important because it may positively or negatively affect the use of wood pastures by woodpeckers.

Landscape variables related to compositional heterogeneity and terrain ruggedness within a radius of 2000 m around the survey point. This radius corresponds to the approximate average valley width of the study area. Birds occurring in farmland mosaics have been observed to respond strongly to landscape heterogeneity [26]. Compositional heterogeneity was calculated as the standard deviation of the monochromatic channel of SPOT 5 data (©CNES 2007, Distribution Spot Image SA). Terrain ruggedness was calculated as the standard deviation of the elevation. It indicated the geomorphology of the surrounding landscape, and also functioned as a proxy for forest cover because highly rugged landscapes tended to be densely forested.

Prior to modelling we log transformed percent woody vegetation within 300 m of the survey point, as well as area of the wood pasture; we standardized all variables by subtracting their mean and dividing by their standard deviation; and we confirmed that variables were not correlated. We then used an information theoretic approach for model selection to identify models that best explained woodpecker presence [27]. We constructed eight alternative candidate models arising from all possible combinations of the three groups of variables (I, I+L, I+B, I+L+B, L, L+B, B) and the null model. We used the R package ‘AICmodavg’ to rank the candidate models, based on AICc values to account for small-sample bias [27]. Models considered best had an AICc difference (ΔAICc) of less than two from the model with the lowest AICc.

## Results

**Figure 2:**
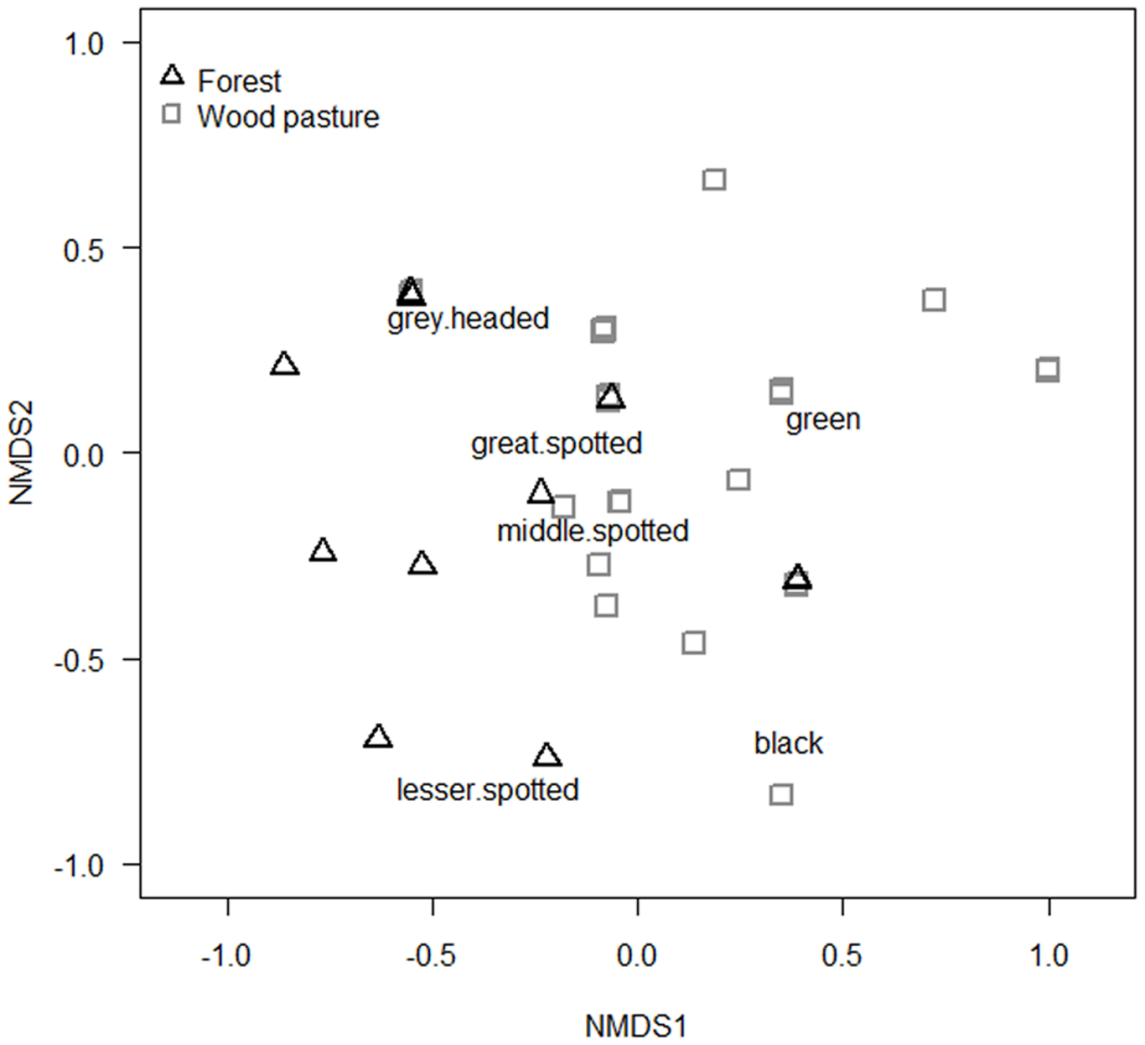
Woodpecker species composition in forests and wood pastures. Non-metric multidimensional scaling of woodpecker composition in both forests and wood pastures, based on a Sørensen dissimilarity matrix (two axes; stress = 15.1).

We found differences in species composition between wood pastures and forests in the ordination and the analysis of similarity (NMDS: two axes, stress = 15.1, see Fig. 2; ANOSIM: R = 0.141, *P* = 0.009). The R-statistic from the analysis of similarity was only slightly larger than zero indicating that compositional dissimilarity between groups was only slightly larger than within groups. Only two species showed a clear habitat preference: the green woodpecker for wood pastures and the lesser spotted woodpecker for forests (Fig. 2). The number of species ranged from two to six in wood pastures, and from zero to four in forests. The mean number of species did not differ significantly between wood pastures (mean: 3.14±0.25) and forests (mean: 2.58±0.38) (GLM, z = 0.91, *P* = 0.34).

We detected the middle-spotted woodpecker in 11, the grey-headed woodpecker in 16, and the black woodpecker in 7 of our 28 wood pasture sites. The best ranked models for individual species included the intermediate context group and the null model for the middle spotted woodpecker, and the intermediate context and broader landscape context groups for the grey-headed woodpecker (Table 2). Within the intermediate context, the amount of woody vegetation within 300 m from the edge of the wood pasture (i.e. a proxy of the amount of perimeter that was forested) had the largest effect on both species, with both responding positively to a more forested perimeter (Table 3). For the black woodpecker only the null model was selected as the best-ranked model (Table 2).

**Table 2:** Full model summary of all the candidate models for the three woodpecker species protected under the EU Bird Directive Annex I.

Species	Model	Log(L)	K	AICc	Δ AICc	W_i_
**Middle spotted**						
**woodpecker**	I*	−15.69	3	38.39	0.00	0.479
	Null*	−18.76	1	39.67	1.29	0.252
	B	−16.86	3	40.73	2.34	0.149
	B+I	−15.05	5	42.83	4.44	0.052
	L	−16.77	4	43.28	4.89	0.041
	L+B	−14.28	6	44.58	6.19	0.021
	L+I	−15.73	6	47.47	9.08	0.005
	L+I+B	−13.94	8	51.46	13.07	0.001
**Grey-headed**						
**woodpecker**	I*	−15.06	3	37.12	0.00	0.606
	I+B*	−13.12	5	38.97	1.85	0.241
	Null	−19.12	1	40.40	3.27	0.118
	B	−18.94	3	44.87	7.75	0.013
	L+I	−14.65	6	45.05	7.92	0.012
	L	−18.05	4	45.54	8.42	0.009
	L+I+B	−12.41	8	48.39	11.26	0.002
	L+B	−17.95	6	51.65	14.53	0.000
**Black woodpecker**	Null*	−15.75	1	33.64	0.00	0.701
	L	−13.91	4	37.56	3.91	0.099
	I	−15.33	3	37.66	4.02	0.094
	B	−15.60	3	38.21	4.56	0.072
	L+B	−12.65	6	41.31	7.67	0.015
	I+B	−14.52	5	41.76	8.12	0.012
	L+I	−13.56	6	43.12	9.48	0.006
	L+I+B	−11.62	8	46.76	13.12	0.001

Best ranked models (Δ_i_ <2) are marked with *.

Model: L = local context; I = intermediate context; B = broader landscape context; Null = null model.

Model summary: Log(L) = the maximised log-likelihood, K = number of estimated parameters; AICc: Akaike’s Information Criterion corrected for small sample bias; ΔAICc: difference in AICc compared with the model with the lowest AICc; W_i_: Akaike weights.

**Table 3:** Model coefficients (and standard errors) of the environmental variables included in the best ranked models in binomial GLMs for the middle spotted and grey-headed woodpecker.

				*Model terms*		
Species	Model	*(Intercept)*	Size wood pasture	Woody vegetation in perimeter	Hetero-geneity	Ruggedness
**Middle spotted**						
**woodpecker**	I	−0.58±0.45	0.40±0.48	1.03±0.52		
	Null	−0.44±0.39				
**Grey-headed**						
**woodpecker**	I	0.34±0.45	−0.25±0.43	1.31±0.55		
	I+B	0.48±0.51	−0.47±0.51	2.41±0.98	0.001±0.51	−1.27±0.72

Model: I = intermediate context; B = broader landscape context; Null = null model.

## Discussion

Our findings highlighted that traditional wood pastures, as well as forests, provide useful habitat for woodpeckers in southern Transylvania. The value of wood pastures was particularly evident for the green woodpecker, which was more likely to occupy wood pastures than forests. This finding is consistent with earlier work by Rolstad et al. [28] who suggested that home ranges of the green woodpecker were often confined to meadows and pastures. The diet of the green woodpeckers consists of ants, and it actively selects foraging sites with a high ant biomass [28], [29]. High ant abundance occurs in grazed semi-natural grasslands [30], whereas forests typically support lower ant abundances than open areas [28]. Furthermore, the green woodpecker avoids foraging in areas with tall and dense vegetation [29]. Thus, the structure and management of wood pastures support optimal foraging habitat for the green woodpecker (and possibly for other species specialized on ants).

The lesser-spotted woodpecker was more strongly associated with forests than wood pastures. Although the home ranges of the lesser spotted woodpecker sometimes include open areas, the species typically avoids open areas for foraging [31]. The lesser spotted woodpecker feeds on insects such as aphids, beetle larvae and ants, which are often found in dead wood [3]. This suggests that wood pastures could provide potential foraging habitat for the lesser spotted woodpecker, and foraging requirements therefore cannot explain why the species appeared to avoid wood pastures. A possible alternative explanation is that the lesser-spotted woodpecker may avoid wood pastures to reduce predation risk. Other authors have suggested that the lesser spotted woodpecker appears to select locations with lower predation risk (e.g. forests compared to more open wood pastures) over locations with higher energetic profitability (Olsson 1998 cited in [31]).

However, wood pastures may be used by other woodpecker species typically associated with forest environments, as demonstrated in Spanish dehesas for the middle spotted woodpecker [15]. Indeed, we found no difference in the number of species between wood pastures and forests, and all three woodpecker species protected by the EU Bird Directive Annex I (and typically considered to be forest-associated) were present in both types of sites.

Surprisingly, environmental variables at different spatial scales had little effect on the presence of protected woodpecker species. The only environmental variable positively related to the presence of two species was surrounding forest cover (especially evident for the grey-headed woodpecker; Table 3). The grey-headed woodpecker may preferentially occupy forest stands containing beech [32], which in our study area more frequently occurred in forests rather than wood pastures. It is possible, therefore, that the grey-headed woodpecker selects nest sites in beech trees within forest patches [23] but uses nearby wood pastures for foraging. We observed the grey-headed woodpecker twice or more in only 9 out of the 22 sites in which it was ultimately detected, which may support the notion that it uses wood pastures for foraging rather than breeding. The grey-headed woodpecker forages on ants, although it is less specialized compared to the green woodpecker [3] – it is primarily a ground feeder when the ground is free of snow or forages on bark-dwelling insects on dead trees during winter [33]. While it appears that wood pastures should provide good foraging habitat for the grey-headed woodpecker, data on breeding locations would be required to further scrutinize this explanation of occurrence patterns.

Similarly to the grey-headed woodpecker, the black woodpecker also may preferentially select beech trees for nesting [23], and its diet also includes ants [3]. However, the black woodpecker is likely to use the landscape at a different spatial scale compared to the other two species. It moves over larger areas [34] and the intermediate spatial scale chosen in our study may have been too small for this species to show an effect of adjacent forest cover.

Local vegetation structure, including the availability of large trees, is known to influence woodpecker presence elsewhere (e.g. [35]). The lack of association of woodpecker species with local variables in our study may reflect that wood pastures contain enough old trees to provide critical habitat elements such as dead wood and food resources [3] - the amount of old-growth elements thus apparently did not limit woodpecker distribution.

Habitat fragmentation by loss of forests has been hypothesized to be one of the major causes of forest bird declines [36]. Because woodpeckers have large home ranges, they may be highly sensitive to forest fragmentation [37], [38]. Given the widespread occurrence of woodpeckers throughout our study area, our results suggest that woodpeckers are likely to perceive the landscape as largely unfragmented: (1) we found no difference in the number of species between forests and wood pastures; and (2) environmental variables such as ruggedness (a proxy for landscape level forest cover) had little effect on the three threatened woodpecker species. Broad-leaved forest cover in our study area is approximately 42%, and existing evidence suggests that fragmentation effects becomes severe only well below this threshold [31], [34], [39]. For example, the black woodpecker was largely insensitive to fragmentation in a highly fragmented landscape with only 26% of forest cover [34]. Moreover, scattered trees are available throughout the agricultural mosaic, very likely providing effective functional connectivity between forest patches [12]. Because we lack information on reproductive performance of woodpeckers in wood pastures compared to forests, we cannot make inferences about the quality of wood pastures as breeding habitat for woodpeckers. Nevertheless, our results suggest that wood pastures provide important feeding habitat for woodpeckers and probably provide connectivity between different forest patches.

### Conservation Implications

Despite their high cultural and natural values, wood pastures are declining rapidly throughout Europe [17]. Considering their important values for a range of species [18], [20], [40], [41], the conservation of wood pastures should be addressed in relevant policies and directives. For woodpecker conservation specifically, we recommend that conservation policies focus not only on maintaining mature forests but also recognize the complementary value of wood pastures that have retained old-growth structures. There are a few national conservation policies for wood pastures (e.g. [42]), but to date there is no pan-European conservation policy [17].

To improve the conservation status of European wood pastures we suggest they should be considered explicitly in two major EU policies: (1) the EU Habitats Directive; and (2) the EU Common Agricultural Policy, specifically with respect to agri-environment payments. Currently, wood pastures are inconsistently considered in the EU Habitats Directive, and the Directive does not include Romanian wood pastures [17]. Although the ecological effect of some agri-environment payments is still debated [43], when appropriately targeted, they can provide a useful tool for farmland biodiversity conservation [44]. In many member states of the EU, agri-environment payments exist for management of extensive pastures, which typically provide subsidies for clearing woody vegetation to maintain extensive grassland environments. However, such payments may inadvertently pose a threat to wood pastures, because scattered tress can fall victim to the clearing process (legally or accidentally, pers. obs.; [45]). More carefully specified agri-environment schemes could stimulate the conservation of trees in extensively managed pastures, thereby recognizing their keystone role for a wide range of organisms [12].
